# Effectiveness and efficiency of assertive outreach for Schizophrenia in Germany: study protocol on a pragmatic quasi-experimental controlled trial

**DOI:** 10.1186/1471-244X-13-56

**Published:** 2013-02-15

**Authors:** Anke Bramesfeld, Jörn Moock, Kirsten Kopke, Dorothea Büchtemann, Denise Kästner, Jeanett Radisch, Wulf Rössler

**Affiliations:** 1 Innovation Incubator, Leuphana University Luneburg, Luneburg, Germany; 2 Institute Epidemiology, Social Medicine and Health System Research, Hanover Medical School, Hanover, Germany; 3 Clinic for Social and General Psychiatry, University of Zurich, Zurich, Switzerland

**Keywords:** Home treatment, Psychiatry, Integrated care, Seriously mentally ill, Controlled trial

## Abstract

**Background:**

A model of assertive outreach (AO) in which office-based psychiatrists collaborate with ambulatory nursing services for providing intensive home-treatment is currently being implemented in rural areas of Lower Saxony, Germany. The costs of the model are reimbursed by some of the statutory health insurance companies active in Lower Saxony. Effectiveness and efficiency of this model for patients suffering from schizophrenia is evaluated in a pragmatic and prospective trial.

**Methods:**

Quasi-experimental controlled trial: patients receiving the intervention are all those receiving AO; controls are patients not eligible for AO based on their health insurance affiliation. Eligibility criteria: clinical diagnosis of schizophrenia (ICD-10 F.20), aged at least 18 years and being moderately to severely impaired in global functioning. Primary outcome: admission and days spent in psychiatric inpatient care; secondary outcomes: clinical and functional status; patient satisfaction with chronic care; health care costs. Follow-up time: 6 and 12 months.

**Discussion:**

The study faces many challenges typical to pragmatic trials such as the rejection of randomisation by service providers, the quality of treatment as usual (TAU) to which the intervention will be compared, and the impairment of the study subjects. Solutions of how to deal with these challenges are presented and discussed in detail.

**Trial registration:**

International Standard Randomised Controlled Trial Number: http://ISRCTN34900108, German Clinical Trial Register: http://DRKS00003351

## Background

The aim of modern mental health care is to enable severely mentally ill people to live integrated in the community and live as autonomously as possible. Modern mental health care does not only address psychiatric and other medical needs but also social and economic ones [1,2]. It is, therefore, usually community oriented and is provided by different professions and services. However, the challenge that arises in modern mental health care is to provide continuity of care across services and service sectors [3,4].

One possibility for improving continuity of care is to provide assertive outreach (AO) treatment. AO is an intensive and highly integrated approach for community mental health service delivery [5]. AO usually includes the following elements: it is provided by multi-professional teams with a psychiatrist integrated into the team; AO takes over responsibility of health and social care; within AO regular and frequent home visits are provided [6]. However, until now, the findings are controversial as to how far AO is better capable of improving patients’ clinical outcomes and to what extent it reduces the need for days spent in hospital compared to TAU [7]. While older studies report significant effects of AO on patients’ wellbeing and days spent in hospital [8,9], more recent studies fail to do so [10,11].

In some countries, AO is being implemented nationwide and long-standing experience with AO does exist, such as in England and in the Netherlands. Other countries, such as Germany or Belgium, do not make AO available or rather, they are just in the process of implementing first AO-style models.

We will discuss a study protocol to evaluate the effectiveness and efficiency of a model of AO that is currently being implemented in rural areas of Lower Saxony, Germany. It is, thus, a protocol not of a trial but of a pragmatic study.

The German mental health care system is known for its fragmentation [12]: there are separate budgets for in- and outpatient care. Most outpatient care is provided by office-based psychiatrists who operate as independent entrepreneurs. Also, ambulatory nursing services operate as independent entrepreneurs. The medical services that are provided by office-based psychiatrists and nursing services are reimbursed within the budget framework of the statutory health insurances. A further problem in mental health care relates to the scarcity of services in rural areas including office-based psychiatrists, outpatient departments linked to hospitals and social services specialised in serving the severely and chronically mentally ill. In this health system environment, possibilities for intensified outpatient care and cooperation across sectors are limited, and incentives to keep patients out of hospital are low. Thus, admitting patients to psychiatric inpatient care occurs rather quickly.

In this situation, office-based psychiatrists in the rural areas of Lower Saxony have set up collaboration with ambulatory nursing services in a framework of integrated care with the aim of providing a model of AO. The model provides intensive and integrated home treatment as an alternative to inpatient admission. The model is meant to serve the more severely ill, particularly persons suffering from schizophrenia. As core components, this model offers the following service elements (see also Table 1):

•Outpatient care is provided by a multi-professional team. The team leader is an office-based psychiatrist.

•An outreach crisis service is available 24 hours, seven days a week. It is operated by ambulatory nursing services and is backed up by an office-based psychiatrist.

•Home -treatment is provided by nursing services and –if needed – by the psychiatrist.

•Case management is provided by the nursing services, coordinating all the care that the patient receives. Every patient is assigned to a specific nurse as her/his personal case manager. This nurse is replaced by other nurses only during times of her/his absence.

•Psychoeducation is offered regularly.

•Regular team conferences are held and attended by at least one representative of the nursing service and the psychiatrist.

•Care is provided on the basis of an evidence-informed treatment pathway. This treatment pathway was developed by psychiatrists participating in the intervention. The treatment pathway is based on the German national guideline for Schizophrenia. It considers regional conditions as well as prior experiences with integrated care concepts. The treatment-pathway defines precisely the interaction between the nursing service and the psychiatrist, but also when and how to involve other services such as social services (that have different cost-carriers and budgets than the medical services), rehabilitation services, or inpatient psychiatric care. The treatment pathway is binding for service providers that participate in the AO model. It provides also the basis for reimbursement of services within the AO-model. Thus for purposes of reimbursement service provision in relation to the treatment pathway is continuously monitored.

**Table 1:** Comparison of service provision in “classic” assertive outreach, assertive outreach as implemented in Lower Saxony and treatment as usual (TAU) in mental health care in Lower Saxony and Germany

**Characteristics of service**	“Classic ” assertive outreach according to DACT-Scale*	**Assertive outreach in Lower Saxony**	**Treatment as usual (TAU) in Lower Saxony/Germany**
Target population	Patients suffering Severe Mental Illness (SMI), high users of mental health services	Patients diagnosed with Schizophrenia	Patients diagnosed with any mental illness
Involved service providers	Multiprofessional: Psychiatrist, nurse and others	Office based psychiatrist in cooperation with specifically trained psychiatric nurse. Further professionals such as rehabilitation specialists are involved as needed. Their involvement is brokered by nurse (case management)	Office based psychiatrist and other services providers as needed (and if available). No standardized pathways for cooperation and exchange between service providers are implemented.
Home treatment	yes	yes	no
Case management	yes	yes	no
Interdisciplinary treatment conferences	yes	yes (usually involving psychiatrist and nurse), meeting once a week	no
Responsible for medical and social needs	yes	yes	no
24/7-service	yes	yes	no
Maximum frequency of contact	high	high	medium
duration of relation	longterm	longterm	longterm

*Dartmouth assertive community treatment scale [13].

Office-based psychiatrists who are interested in offering this type of AO sign up with a management association (IVPNetworks GmbH). This management association takes care that psychiatrists perform according to the treatment guideline and it negotiates specific reimbursement schemes for the intervention with statutory health insurance companies. For the time being, the management association has contracted for AO with the Allgemeine Ortskrankenkasse (AOK) in Lower Saxony and with the Techniker Krankenkasse (TK).^a^ Among the patients served at one office based psychiatrist there can be some patients who are eligible for receiving AO, due to their health insurance affiliation, while others are not. Those eligible have to give written consent to be treated with AO. Currently, about 40 psychiatric practices in Lower Saxony have joined this management association. They provide AO in an area of 16,000 km^2^ and a population of 2 million (of which approximately one third is insured with the AOK or the TK).

The study presented in this protocol tests the following two main hypotheses:

I. Schizophrenic patients who are served by AO have significantly fewer admissions to psychiatric inpatient treatment than patients receiving TAU.

II. Schizophrenic patients who are served by AO have significantly fewer days of psychiatric inpatient treatment than patients receiving TAU.

In addition, further hypotheses are as follows:

III. Schizophrenic patients being served by AO do significantly better clinically and functionally than patients receiving TAU.

IV. Schizophrenic patients being served by AO are significantly more satisfied with the chronic care they receive than patients receiving TAU.

V. Treating schizophrenic patients by AO is more cost-effective than treating schizophrenic patients by TAU.

## Methods

The hypotheses named above will be tested in a prospective and controlled cohort study that is quasi-experimental due to the patient´s health insurance affiliation. The intervention of AO will be tested against TAU. Follow-up occurs after six and 12 months.

### Outcome and process parameters

The main outcome parameters are the number of admissions to inpatient treatment as well as the number of days spent there. Further outcome parameters include: psychiatric symptomatology, functional status, life satisfaction, adherence to medication, substance misuse, use of services, and satisfaction with chronic care. For details of the scales that will be used to assess these outcome parameters, please see Table 2.

**Table 2:** Instruments to assess and process

**Outcome parameter**	**Instrument**	**Hypotheses**	**To be filled out by**
**Psychosocial functioning (inclusion criteria)**	Global assessment of functioning [14]	II	Psychiatrist
**Psychopathology**	Brief Psychiatric Rating Scale (BPRS) [15]	II	Psychiatrist
			Patient
**Life satisfaction**	11 point scale from the German Socioeconomic Panel [16]	II	Patient
**Functional impairment**	WHO-Disability Assessment Scale (WHO-DAS II) [17]	II	Patient
**Substance misuse**	Alcohol Use und Drug Use Scale (AUS, DUS) [18]	II	Patient
**Medical adherence**	Medical Adherence Rating Scale (MARS) [19]	II	Patient
**Patient satisfaction with medical care**_**1**_	Patient Assessment of Chronic Care (PACIC) [20]	III	Patient
**Service use**^**1,2**^	Client Sociodemographic and Service Receipt Inventory (CSSRI) [21]	I+IV	Practice assistant

^1^ Scales assess outcome as well as process: receiving care in concordance with the chronic care model (PACIC) and use of services (CSSRI).

^2^ Is assessed at t0 and then every three months, in contrast to all other parameters that are assessed at t0 and six and 12 months later.

When selecting scales to measure outcome parameters, we did not only consider specificity and reliability of instruments but also practicability. We expect that the patients, who will be recruited into the study, will be quite impaired by the illness. Therefore, questionnaires need to be simple, easy to handle and not too time-consuming.

All parameters will be assessed at baseline (t0), after six months (t1), and after 12 months (t2) from all patients receiving the intervention and from all controls. The use of services however will be assessed every three months (5 times) from both groups.

The use of services will be assessed by means of the German version of the Client Sociodemographic and Service Receipt Inventory (CSSRI; [22]). The CSSRI assesses the use of services retrospectively for the past three months by questioning the patient. The CSSRI reports not only the quantitative use of inpatient and outpatient medical and psychiatric services and medication, but also the use of other forms of public support. This includes the use of social services, such as housing, rehabilitation services, the patient’s income, including social benefits and other forms of state payments, as well as their vocational affiliation and number of days of sick leave. Thus, the CSSRI assesses not only the main outcome, i.e., days spent in psychiatric inpatient care, but also serves as documentation of the process of care across sectors and services for both the intervention and the control group. Finally, the information gathered by the CSSRI allows for calculating direct as well as indirect health care costs [22,23].

Likewise, the German version of Patient Assessment of Chronic Care (PACIC; [24]) that is used to measure patients´ satisfaction with chronic care, serves as an instrument to measure outcome as well as process. The PACIC assesses whether patients receive specific services and information – such as health education and shared decision making – that are proven to be significant in the care of chronic patients [25]. Originally, the PACIC was developed for somatic medicine. However, it has been shown to be relevant also in the evaluation of care provided to mentally ill patients [26].

### Recruitment

Patients will be recruited in the practices of office-based psychiatrists who provide AO. Recruitment will be done by the treating psychiatrist. Unfortunately, we were not successful in convincing the attending psychiatrists to support “real” randomisation of patients receiving AO or those receiving TAU. “Real” randomisation would have meant that the office-based psychiatrists would lose 50% of the extra money they receive from health insurance companies when providing AO. Therefore, a quasi-experimental design will be used instead. As patients allocated to receive the intervention all those patients are considered, who are insured by a health insurance that reimburses AO, and who in fact receive AO. All patients who - due to their health insurance affiliation - are not eligible for AO are considered to be controls. We assume that the affiliation with a specific statutory health insurance company is more or less random. Choice of health insurance company is free, all statutory health insurance companies offer approximately the same services, cost the same and are obliged to insure people regardless of their health and financial status. Even if there might be some differences in social status between the members of different health insurance companies, these differences are most likely obliterated by the social consequences of suffering from schizophrenia.

In this quasi-experimental design, all attending practices recruit the same number of patients receiving the intervention and of patients being controls. An honorarium of 150€ will be paid to the practice per assessment and patient. Patients will receive an honorarium of 20€ per assessment.

Eligibility criteria are: being at least 18 years of age, suffering from schizophrenia (ICD-10 F 20) as diagnosed clinically by the treating psychiatrist, having a maximum score of 60 on the Global Assessment of Functioning Scale (GAF) [14] and being cognitively and linguistically able to fill out the patient questionnaire.

Recruitment is planned to take a year.

### Preparing and assisting data collection in the practices

The methodology of the study was developed in close exchange with the psychiatric practices of the management association providing AO. To make this pragmatic trial [26] work, we sought to adjust our study design as closely as possible to the conditions and requirements of daily life and work in the psychiatric practices. Further, all practices are visited by a member of the research team for recruitment and for explaining the study. This team member then becomes the personal contact for this practice throughout the study. The team member trains the practice assistant in handing out and collecting the study materials to patients receiving the intervention and to controls. During recruitment and assessment times the study-team member holds weekly telephone contact to its assigned practice. A telephone hotline is implemented for upcoming urgent questions concerning the study.

### Sample size

Previous studies performed in Leipzig, Germany, have shown a mean number of days spent in psychiatric inpatient care by schizophrenic patients of 47 (SD 83) [27]. Further, studies estimated the mean decrease of inpatient days that can be expected from interventions to be 40% [28]. According to Hedeker and colleagues (1999), a baseline sample size of 242 participants in each group would be required to detect a small effect size of 0.2 with a power of 0.80 at a two-tailed significance level of 0.05 [29].

Previous studies have shown that 20% of patients cannot be traced for follow-up or do not return questionnaires over a 12-month survey period [27].We aim to reduce drop-out numbers by keeping records of patients' telephone contact details as well as secondary contact names and addresses at the practices of the attending psychiatrists. If patients fail to return for assessment to the attending practice, they are contacted by phone and asked whether they want to continue with the study. This way, we hope to keep the number of drop-outs at a rate of 10%. Thus, the total number of participants to be initially recruited will be 268 patients per group or 536 in total.

### Non-response

Even in case of low rates of non-response, patients who are lost to follow-up represent a potential source of selection bias. Therefore we will present a flow chart containing the numbers of patients in different stages of the study (e.g. screened for eligibility, allocated, follow-up) and report reasons for non-response. Furthermore, we will compare baseline characteristics of non-responders and study participants (age, gender and assessment of functioning) as well as of those who are lost to follow-up and those who retain.

### Statistical analysis

We will analyse data using per-protocol analysis, considering only those patients who successfully completed the study, as well as using an intention-to-treat approach. Thereby we would also consider those patients who dropped out of the study or changed their originally allocated group.

We will report baseline demographic and clinical characteristics for each study condition and test for baseline equivalence using parametric and non-parametric test as appropriate. If necessary, we will control for baseline differences. Missing data will be analysed.

Longitudinal data will be analysed using standard models for repeated measures including multivariate mixed effects analysis of variance, random effects and generalized estimating equation (GEE) models, as appropriate.

Whenever possible, measures of "practical relevance" such as proportion of explained variance, accuracy of prediction, or effect size will be reported. Statistical analyses will be performed using standard software packages (SPSS, STATA).

### Ethics and dissemination

Written consent is sought from all participants. For those under legal guardianship, consent is also sought from the guardian. The patients’ identity on the questionnaires will be pseudonymised by the recruiting practice. Only the pseudonymised filled-out questionnaires will be sent to the study centre. Thus, there is no direct contact between patients and researchers. The Research Ethics Committee of the Lower Saxony Medical Association approved the study (Ärztekammer Niedersachsen BO/03/2011). It is compliant with the Helsinki Declaration. The reporting of the study will be in accordance with standards of the CONSORT extension for pragmatic trials and the TREND statement [30,31].

## Discussion

There are many challenges confronting this pragmatic study on the effectiveness and efficiency of this assertive outreach (AO) model in rural areas of Lower Saxony in Germany:

### Researching a real life implemented intervention

While it is common in trials that extra time is planned for the tasks related to research, this is not the case in this pragmatic study evaluating a real-life implemented intervention. All service providers participating in the study do so voluntarily and in addition to their daily routine and regular professional obligations. So first of all, office-based psychiatrists have to be convinced to participate in the study. Then, they have to recruit patients for participating in the study, seek their consent, hand them questionnaires, fill in questionnaires themselves and send pseudonymised data to the study centre. To motivate the psychiatrists to do all these additional tasks in conjunction with the study, they are offered quite a generous honorarium. Further, all practices will have a personal researcher assigned who will support them with advice during the whole process of the study. In addition, the study protocol seeks to keep extra work for service providers to a minimum. Thus, most questionnaires are filled out by the patients themselves. The questionnaire on use of services needs to be filled out by the practice assistant together with the patient.

Despite the challenge of motivating office-based psychiatrists to participate in the study and of keeping their motivation high during the course of the study, a pragmatic study has advantages over an experimental trial. While a trial is always a kind of in vitro artificial situation, a pragmatic study deals with the real life conditions of the health system. This implies that patients appear with the average mix of co-morbidities. Also, health care providers have, on average, a mixture of qualifications compared with top-level trained specialists in academic centres. They all operate within the constraints of the health system. These real life conditions may make research more difficult however, they also increase external validity [32]. In case that a real life study succeeds in showing the effectiveness and efficiency of an intervention, the likelihood increases that this intervention does in fact work [33]. On the other hand, the validity of the results is impaired by possible selection biases, as in the study presented here, real randomisation was not possible. At the same time, the – possibly effective – intervention is likely to be more sustainable than interventions that are only implemented in the context of a trial. Many home treatment interventions that were implemented in the context of a trial were not maintained for long beyond the trial [34].

### Researching severely ill people

AO is meant to be an intervention for the more severely mentally ill. To assure this, the Global Assessment of Functioning Scale (GAF) is used as an inclusion criterion. This means that patients in this study experience at least moderate to severe problems in social or vocational functioning. We expect many patients to be much more impaired than GAF 60. As our pre-tests showed, filling out a questionnaire can be quite challenging to such impaired patients. To enable a broad participation also for more severely ill persons, we had to keep the questionnaires as lean as possible. This implies that we preferred shorter, simplified questions over the original longer ones. We boiled down quality of life to one question rating life satisfaction on an 11 point scale [16] as well as restricted the alcohol and drug use scale to two introductory questions referring to the quantity of alcohol and drug intake.

### Controlling for TAU

Whether an intervention that changes the modes of services provision such as case management is effective or not, is not only related to the strength and quality of the intervention, but also the quality of TAU to which the intervention is compared is decisive [35,36]. This study will assess the use of services in intervention and TAU condition every three months by means of the Client Sociodemographic and Service Receipt Inventory (CSSRI). Thereby, we will be able to describe in detail characteristics and differences in the care that patients receive in the intervention and TAU group. This will enable us to interpret our outcome findings by considering the process of care.

### Bias

We aim to recruit equal numbers of patients receiving the intervention and of controls from the same psychiatrist. Thus, possible bias in respect to context variables will be minimised. Further, the relatively high number of participating psychiatrists should eliminate data distortion due to different physician-patient interaction styles.

The quasi-experimental design that distinguishes participants by their affiliation to a specific health insurance company and thus, their eligibility for AO, can limit the study validity. The data from the study will show whether our assumption is true that schizophrenic patients in different health insurance companies are more or less equal in terms of socio-economic and health status.

## Conclusion

In a mental health system that is as fragmented as the German one, it is crucial to find interventions that bridge some of the fragmentation in service provision. The model of AO that is to be researched in this study presents one possible solution to some of the fragmentation of the system. It is innovative in the context of the existing system. By integrating outpatient psychiatric care with ambulatory nursing services, the model provides an AO-style intervention to the severely mentally ill. The findings from the study, regardless of whether they will support or reject the hypotheses, will be of great use for the further development of integrated care and home care in Germany’s mental health system, as well as in other similar health systems.

## Endnotes

^a^There are 146 statutory health insurance companies in Germany, insuring approximately 90% of the population. AOK and TK are among the larger health insurance companies. AOK Lower-Saxony insures more than one quarter of the population in Lower-Saxony, TK insures close to one tenth of the population in Germany. While AOK insured traditionally have a lower socioeconomic status, TK insured come from an academic and middle class background. Probable social disparities between the insured populations of health insurances companies are originated in former times, when health insurance affiliation was bound to the workplace. For more than 15 years now, choice of health insurance has been free and social disparities between the insured populations of the health insurance companies are levelling out.

## Competing interests

There are no conflicting interests of any of the authors in respect to this study.

## Authors’ contributions

AB participated in the development of the study design and drafted the paper. JM drafted the methods section and critically revised the other sections. KK participated in the development of the study design and in drafting the paper. DB participated in the development of the study design, in particular the selection of instruments, and revised the paper critically. DK participated in the development of the study design, in particular the selection of instruments, and revised the paper critically. JR participated in the development of the study design and revised the paper critically. WR authored the study design, applied for the funding and revised the paper critically. All authors read and approved the final manuscript.

## Pre-publication history

The pre-publication history for this paper can be accessed here:

http://www.biomedcentral.com/1471-244X/13/56/prepub
